# Crime: between symptom and trauma

**DOI:** 10.1192/j.eurpsy.2025.231

**Published:** 2025-08-26

**Authors:** F. Boaron

**Affiliations:** Dipartimento ad attività integrata Salute Mentale e Dipendenze Patologiche, AUSL Reggio Emilia, Reggio Emilia, Italy

## Abstract

**Abstract:**

Exposure to Adverse Childhood Experiences (ACEs) represents a significant, dose-dependent risk factor for the development of numerous psychiatric and physical disorders (including diabetes, myocardial infarction, obesity, alcoholism, drug addiction, etc.), as well as for poorer social outcomes (dropping out of school, unemployment, economic decline, unwanted pregnancies) and violent behaviour.

A history of maltreatment is found in 80–90% of young offenders, and about one quarter of individuals with a history of severe maltreatment receive criminal convictions. Among the forensic psychiatric patients admitted to the REMS (Residential Facilities for the Enforcement of Security Measures), we have likewise observed a high prevalence of maltreatment, abuse, neglect, and other traumatic events, including those related to war and migratory pathways.

We have treated some patients showing psychotic-spectrum symptoms (delusions, hallucinations) that did not respond to pharmacological treatment (including clozapine and clozapine–first-generation antipsychotic combinations). Their condition improved only when trauma-oriented psychotherapy, such as EMDR or Narrative Exposure Therapy for Forensic Offender Rehabilitation (FORNET), was added to antipsychotic treatment.

This observation raises critical questions about the overlap between psychotic and post-traumatic symptoms in offending patients with severe early victimization histories, and hence about the link between schizophrenia spectrum disorders and complex post-traumatic stress disorder. In light of ongoing conflicts, rising geopolitical tensions, and their impact on migration, the early diagnosis and effective treatment of these conditions are of paramount importance from both a public health perspective and from the standpoint of security and the prevention of violent offenses

**Disclosure of Interest:**

None Declared

